# Supplementary figures and images for: SNP Data Quality Control in a National Beef and Dairy Cattle System and Highly Accurate SNP Based Parentage Verification and Identification
Source: Front Genet. 2018 Mar 15;9:84. doi: 10.3389/fgene.2018.00084 (PMC5862794; doi:10.3389/fgene.2018.00084)

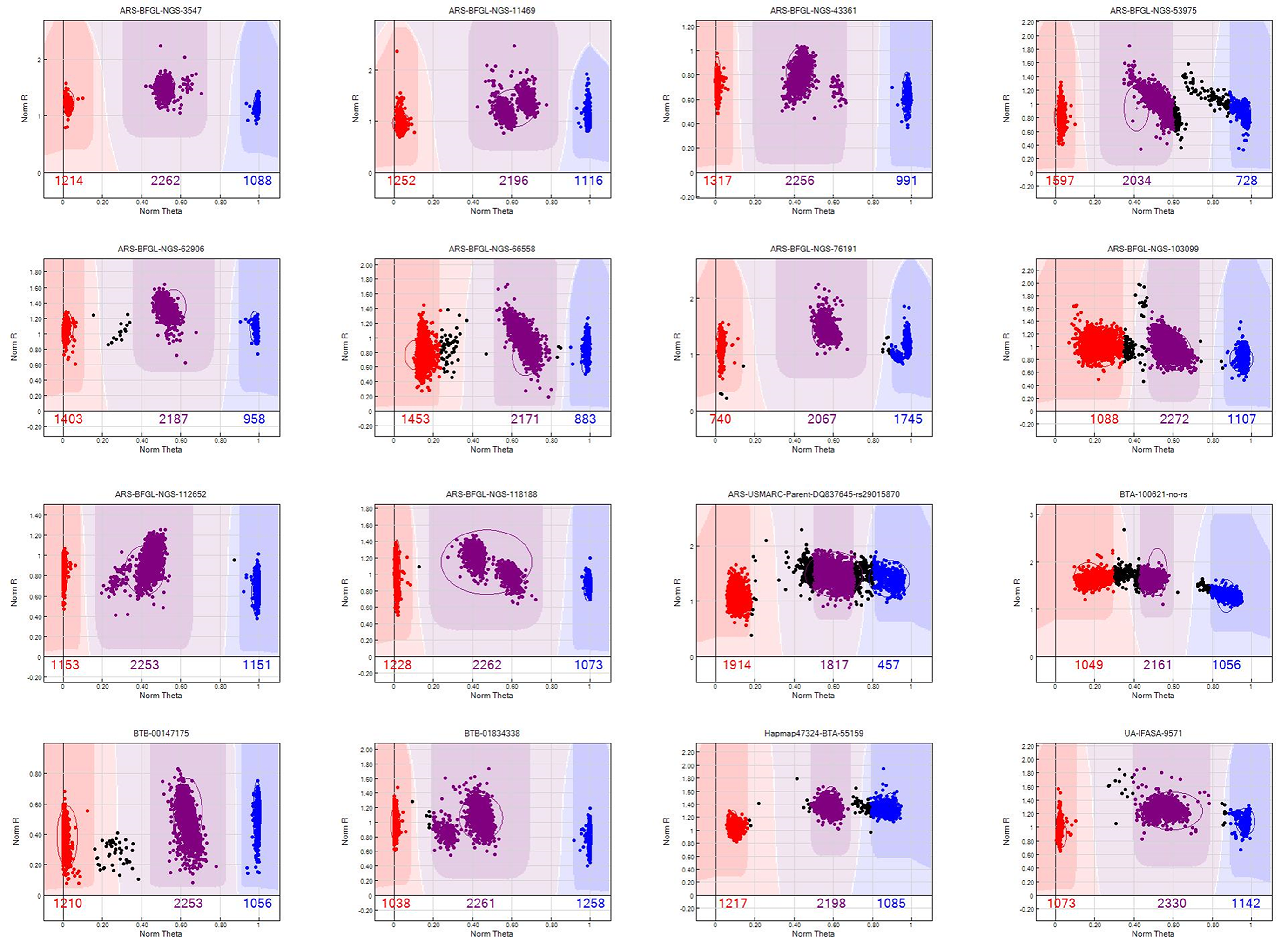

Supplement: Figure S1 — SNP cluster issues for 16 SNP based on Illumina GenomeStudio 2.0 SNP cluster plots of 4,561 animals from multiple breeds. SNP ID (Left to Right and Top to Bottom) are: ARS-BFGL-NGS-3547, ARS-BFGL-NGS-11469, ARS-BFGL-NGS-43361, ARS-BFGL-NGS-53975, ARS-BFGL-NGS-62906, ARS-BFGL-NGS-66558, ARS-BFGL-NGS-76191, ARS-BFGL-NGS-103099, ARS-BFGL-NGS-112652, ARS-BFGL-NGS-118188, ARS-USMARC-Parent-DQ837645-rs29015870, BTA-100621-no-rs, BTB-00147175, BTB-01834338, Hapmap47324-BTA-55159, UA-IFASA-9571. [file Image1.TIF]
